# Can Hotel Companies’ Water Conservation Management and Waste Reduction Measures Influence Hotel Customers’ Willingness to Pay More and Intention to Revisit?

**DOI:** 10.3390/ijerph18179054

**Published:** 2021-08-27

**Authors:** Junghyun Park, Yunmi Park, Jae Leame Yoo, Jongsik Yu

**Affiliations:** 1 College of Hospitality and Tourism Management, Sejong University, 98 Gunja-dong, Gwanjin-gu, Seoul 05006, Korea; junghyun.peterpark@gmail.com; 2 Department of Aviation Service, Cheongju University, 298 Daesung-ro, Cheongwon-gu, Cheongju-si 28503, Korea; ympark@cju.ac.kr; 3 Department of Aeronautical Science and Operation, Cheongju University, 298 Daesung-ro, Cheongwon-gu, Cheongju-si 28503, Korea; jlyoo@cju.ac.kr; 4 College of Business Division of Tourism and Hotel Management, Cheongju University, 298 Daesung-ro, Cheongwon-gu, Cheongju-si 28503, Korea

**Keywords:** water conservation management, waste reduction measures, social norm, personal norm, willingness to pay more, revisit intention, cost consciousness

## Abstract

This study investigated the effect of hotel water conservation management and waste reduction measures on customers’ social and personal norms, willingness to pay more, and revisit intention, with cost consciousness as a moderating variable. A total of 311 valid samples were obtained by conducting a survey on customers who have used hotels for the past year. To perform the empirical analysis, SPSS 22.0 (IBM, New York, NY, USA) and AMOS 22.0 (IBM, New York, NY, USA) were used. As a result of the analysis, seven of the eight hypotheses were accepted, and the ninth hypothesis that tested the moderating effect was partially accepted. The results of the study revealed that a hotel’s eco-friendly activities had a positive effect on its overall performance. The results also provide insight that can lay the foundation for the sustainable management of hotels.

## 1. Introduction

The growth of the hotel industry has both positive and negative effects. It provides various benefits to consumers and equally causes environmental problems. More specifically, the hotel industry provides its guests with conveniences such as accommodation, food, banquets, and performances, which improve consumers’ living standards. Conversely, wastes generated by the hotel industry such as water pollution, household waste, greenhouse gas, and food waste can be a major source of environmental pollution [1]. Specifically, hotels generate 289,700 tons of waste each year (including 78,000 tons of food waste), and restaurants generate 915,400 tons of waste annually, including 199,100 tons of food waste [2]. In addition, wastewater from bathing, laundry, and toilets are generated in hotels through guest use. Furthermore, wastewater from food preparation, cleaning, swimming pool, and spa are generated in the process of operating the hotel [3]. Because of the large amount of waste generated, the hotel industry is causing serious environmental harm and problems.

The modern hospitality industry attaches a great deal of importance to eco-friendly activities and environmental sustainability [4]. Emphasis on the importance of eco-friendliness in the hospitality industry and its sustainability is due to strong consumer demands for eco-friendly products and services [4,5]. This change in consumers’ perception of the environment is because they witness the damage posed by environmental degradation, and they continue to demand measures to minimize pollution and take environmentally responsible actions [6]. A study by TripAdvisor found that travelers are concerned about environmental issues when making hotel decisions [5,7]. Consequently, consumers’ concerns and interest in the environment can influence hotel companies’ environmental protection measures and sustainable development, which can be a good strategy for maximizing positive customer behavior and performance for companies. 

As various problems arise owing to the wastes (e.g., food, plastics, disposable items, etc.) generated in service industries such as hotels, many studies have been conducted on eco-friendly activities and consumer behavior [8,9,10]. However, despite studies on eco-friendliness, research on the complex relationship between a hotel’s eco-friendly policies and consumers’ hotel-oriented behavior is currently underexplored. Therefore, new concepts of water conservation management and waste reduction measures are presented to expand existing research on hotel eco-friendly activities in this study. In addition, this study provides a complex process for explaining hotel performance. To address the purpose of this study, the following aspects are investigated: (1) the effect of conservation management and waste reduction measures on social and personal norms, (2) the effect of social and personal norm measures on willingness to pay and revisit intention, and (3) the moderating role of cost consciousness and its relationship between the presented variables.

## 2. Literature Review 

### 2.1. Water Conservation Management and Waste Reduction Measures

Environmental pollution (e.g., carbon emissions, fine dust, water pollution, solid waste, etc.) occurs when companies produce products and services. In particular, hotels generate a large amount of wastewater and other waste products. Products provided to customers in hotels are divided into guestroom products and restaurant products, and a large amount of waste is generated to maintain or produce them. Specifically, a large amount of water is used in the process of washing bed sheets, towels, pillows, and the like, which constitute the guestroom products. When customers use the toilet and bathroom, a large amount of wastewater is generated [11]. In addition, hotel rooms contain plastics (e.g., water bottles, toothbrushes, toothpaste, shampoo), glass (e.g., various liquor bottles), plastic bags (e.g., laundry covers, disposable cases), and cans (e.g., beverage cans). The same variety of waste by water use and wastewater is also generated in hotel restaurants. In hotel restaurants, a large amount of water is used in the process of cooking the food served to customers. In this process, wastewater and food waste are generated [12]. Physical, chemical, and biological processes are used to manage the reduction of unpleasant and hazardous characteristics of large amounts of waste generated [3]. As such, the amount of water consumption and wastewater generation in hotels is at a level that cannot be ignored and is much likely to continue to increase in the future [6,13]. Furthermore, it has the potential to pose a very high risk to human life by causing environmental problems such as global warming, fine dust, carbon emissions, and water pollution [14].

Water conservation and waste reduction measures are being used as important green management strategies worldwide [5,15,16]. Many companies use green management strategies such as water conservation and waste reduction measures because customers are more concerned about the environment than ever before. This allows customers to believe that their attitude in using products and services can contribute to environmental problems, and it can be assumed that other people also have these beliefs [17]. Therefore, customers can monitor corporate activities that may cause environmental problems, and it is much likely that they expect companies to do eco-friendly activities. Joshi and Rahman [18] reviewed 53 papers on empirical studies related to green purchasing intentions and behaviors from 2000 to 2014. The results revealed that consumers’ environmental concerns and functional attributes of products and services were related to consumers’ green purchasing intentions and behaviors. It has been shown to have a positive effect on behavior. In other words, consumers’ environmental concerns and functional properties of products and services can have a positive effect on shaping social and personal norms. According to a study by Matthies et al. [19], waste management by a company to prevent environmental pollution can create a positive attitude toward the company, which can impact the formation of personal norms through such a relationship. In addition, social and personal norms highly motivate one’s behavior when activated by external factors [8]. Consequently, it can be seen that eco-friendly activities such as water conservation and waste reduction measures in hotels play a very important role in the formation of social and personal norms of consumers. Therefore, this study proposes the following hypotheses based on previous studies:

**Hypothesis** **1** **(H1).**  *Water conservation management will have a positive effect on the social norms.*

**Hypothesis** **2** **(H2).**  *Waste reduction measures will have a positive effect on the social norms.*

**Hypothesis** **3** **(H3).**  *Water conservation management will have a positive effect on personal norms.*

**Hypothesis** **4** **(H4).**  *Waste reduction measures will have a positive effect on personal norms.*

### 2.2. Social and Personal Norms

Several studies on environment explain that social norms are an essential concept in shaping consumers’ eco-friendly intentions and determining behaviors [19,20,21]. Social norms refer to the level of social pressure of an individual to perform or not to perform a specific behavior in a specific situation [21,22]. In addition, the concept of social norms is a key component of behavior and motivation and can be defined as an important factor that can influence and change behavior [23]. Social norms are a useful predictor that can foretell individual intentions and behaviors in the social/environmental literature [24]. When individuals perform certain behaviors, they experience social norms. In particular, it has been shown that social norms have a great influence on eco-friendly behaviors and decisions [17,24]. According to Song et al. [25], social norms play an essential role in establishing behavioral intentions for repurchasing tourism products. Gossling et al. [26] found that social norms positively elicit fine dust reduction behaviors, energy saving, and eco-friendly behaviors, which in turn led to an individual’s eco-friendly behaviors (e.g., active purchasing behavior to products and services of companies active in eco-friendly policies and actions).

Personal norms refer to the belief that an individual’s particular behavior is appropriate or inappropriate [27,28]. In addition, personal norms mean a sense of duty to make certain actions or decisions according to one’s own principles or beliefs, which is highly likely to be beneficial to public goods [29]. In other words, it can be said that personal norms shape an individual’s altruistic and socially responsible intentions and behaviors [30]. These personal norms are closely related to a company’s eco-friendly activities. According to Kim and Seock [17], it is explained that when personal norms are formed, purchasing behavior for a specific company’s products and services appears more actively. Doran and Larsen [31] argued that personal norms have a positive effect on eco-friendly behavior, which can lead to intentions and behaviors to purchase eco-friendly products and services. In other words, the personal norm is an appropriate variable to explain and predict eco-friendly behavior [32]. Therefore, the following hypotheses are established:

**Hypothesis** **5** **(H5).**  *Social norms will have a positive effect on willingness to pay more.*

**Hypothesis** **6** **(H6).**  *Personal norms will have a positive effect on willingness to pay more.*

**Hypothesis** **7** **(H7).**  *Social norms will have a positive effect on revisit intention.*

**Hypothesis** **8** **(H8).**  *Personal norms will have a positive effect on revisit intention.*

### 2.3. Willingness to Pay More and Revisit Intention

Willingness to pay more refers to the maximum monetary value that consumers are willing to spend in the process of acquiring a specific product or service [33,34]. Consumers are more active in purchasing products and services when their preferred brands or corporate policies are in line with their beliefs. In particular, as concerns about the environment are increasing, consumers’ willingness to pay more for eco-friendly products and services continues to increase, and eco-friendly products and services are sold at a higher price than other products [35,36]. Lee et al. [37] found that consumers are willing to pay more when purchasing green products and services. Agag et al. [38] also confirmed that consumers are willing to pay more for green products. Therefore, to maximize a company’s performance, it is necessary to persuade consumers to pay more through eco-friendly activities.

To maximize the company’s performance, customers’ revisits are essential. For companies, it is more effective in terms of the time and cost required for customers to repurchase their products and services than to acquire new customers. Therefore, customers’ revisit is a very important factor for generating profits and future growth of the company, and it is perceived as an important success factor of every company [39]. Consequently, the importance and necessity of revisit have been emphasized in many studies because customer revisit plays an absolutely important role in corporate performance [40,41,42]. In particular, many studies have demonstrated a causal relationship between a company’s eco-friendly activities and customers’ intention to revisit. Han et al. [5] found that hotels’ eco-friendly activities, such as water conservation and waste management, had a positive effect on the formation of hotel customer loyalty. Considering the results of this study, it can be said that the customer revisit is an essential element for the company’s performance, which can only happen with a continuous effort to build the relationship between the company and the customer. 

### 2.4. Moderating Role of Cost Consciousness

Cost consciousness is one of the factors that consumers consider when choosing products and services. Therefore, cost consciousness can be perceived as an important consideration in predicting the behavior of many people with rational economic activities [43]. Looking at some previous studies, cost consciousness was defined as the degree of concentration when purchasing inexpensive goods and services [44,45,46,47]. In general, price-sensitive customers want to pay a lower price and tend to seek psychological and financial benefits from price comparison [44]. Conversely, sensitivity to price may appear differently depending on the degree to which ones they consider as important or valuable. In particular, cost awareness can affect consumer behavior in environmental issues such as wastewater management, carbon emissions, and waste management. According to Bai and Bai [43], it was argued that cost awareness moderates environmental commitment and environmental protection actions. Schroder [47] also argued that cost awareness can cause a conflict between social norms or social values and consumer behavior, as cost awareness is related to the sensitivity to the cost. That is, the intention to pay a slightly higher cost or the willingness to purchase again despite paying a slightly higher cost may vary depending on the degree of awareness on cost. Therefore, the following hypotheses are proposed:

**Hypothesis** **9** **(H9a).**  *Cost consciousness is likely to moderate the relationship between social norms and willingness to pay more.*

**Hypothesis** **9** **(H9b).**  *Cost consciousness is likely to moderate the relationship between personal norms and willingness to pay more.*

**Hypothesis** **9** **(H9c).**  *Cost consciousness moderates the relationship between social norms and revisit intention.*

**Hypothesis** **9** **(H9d).**  *Cost consciousness moderates the relationship between personal norms and revisit intention.*

## 3. Methods

### 3.1. Measurement Instruments

Measurement items that have been confirmed reliable and valid in previous studies [5,21,36,42,48,49,50,51] were adapted for this study, and 23 of them were used. Specifically, four question items, including ‘This hotel completely uses low flow toilets and good sanitation practices,’ were used to measure its water conservation management. Four question items, including ‘This hotel completely uses recycled materials (e.g., paper, plastic),’ were used to measure its waste reduction measures. Three question items, including ‘Most people who use the hotel should participate in waste reduction and recycling while staying at the hotel,’ were used to measure the social norm. Three question items, including ‘I feel obligated to reduce and recycle waste while using the hotel,’ were used to measure the personal norm. Three question items, including ‘I am willing to pay more for this hotel,’ were used to measure the willingness to pay more. Three question items, including ‘I will continue to visit this hotel in the future,’ were used to measure revisit intention. In addition, three question items, including ‘I take environmental protection and energy-saving actions to save money,’ were used to measure cost consciousness. The questionnaire comprised a description of the study, questions about measurement variables, and demographic characteristics of the respondents. Most commonly used multi-items were used and all measurement items were rated on a 7-point Likert scale ranging from 1 (strongly disagree) to 7 (strongly agree). The questionnaire was modified through preliminary surveys of the following group of respondents: hotel staff with more than 10 years of work experience, graduate students specializing in hotel management, and university professors who studied hotel tourism. It was finally confirmed through a review of three hotel and tourism experts.

### 3.2. Data Collection and Sample Characteristics

In this study, an online research firm based on a web-based system was used to collect data for empirical analysis. The convenience sampling method was adopted, and it was designed so that all respondents could participate voluntarily after reading the purpose of the study. In addition, to achieve the purpose of this study, questionnaires were administered to customers who have used a hotel for the past year. A screening question to confirm the hotel experience was included in the questionnaire. An empirical analysis was performed based on a total of 311 samples obtained. Regarding the characteristics of the sample, 152 (48.9%) were male and 159 (51.1%) were female. As for age, 74 respondents were in their 20s (23.8%); 159, in their 30s (51.1%); 50, in their 40s (16.1%); and 28 in their 50s or older (9.0%). In terms of educational background, four respondents (1.3%) had a high school diploma; 89 (28.6%), a vocational college degree; 190 (61.1%), a college degree; and 28 (9.0%), a graduate degree or higher. Lastly, the annual income of 81 respondents (29.2%) was less than $30,000; 109 respondents (35.0%), from $30,000 to less than $50,000; 66 respondents (21.2%), from $50,000 to less than $70,000; 15 respondents (4.8%), more than $70,000 and less than $100,000; and 30 respondents (9.6%), more than $100,000. 

## 4. Results

### 4.1. Measurement Model Results

The most suitable methods for this study, namely confirmatory factor analysis, is used to verify the reliability and validity of the scale. The results of the confirmatory factor analysis are as follows. First, the model fit of the measurement model presented in this study is χ^2^ = 454.623, df = 209, *p* < 0.001, χ^2^/df = 2.175, RMSEA = 0.062, CFI = 0.970, TLI = 0.963, indicating that it is statistically significant. The level of model fit is confirmed. Next, the average variance extracted (AVE) and composite reliability (CR) are shown to prove the central validity and internal consistency of measurement items. An AVE value of higher than 0.5, and a CR value over and above 0.7 are said to confirm the internal consistency and central validity of the measured variables [52]. Looking at the analysis results, the CR values of the measured variables in this study range from 0.838 to 0.937, and the AVE values range from 0.636 to 0.793. Therefore, it can be said that there is no problem in the concentration validity and internal consistency of the measurement variables. Next, the discrimination validity is verified to confirm the differentiation between the constructs. It can be verified by comparing the AVE value and the square value of the correlation coefficient. If the AVE value is greater than the square value of the correlation coefficient, the discrimination validity is secured [52]. Examining the results of the analysis, the square value of the correlation coefficient presented in this study is higher than the AVE value. Therefore, there is no problem in the discrimination validity between the constructs presented in this study. The detailed analysis results are shown in Table 1.

### 4.2. Structural Model Results and Hypotheses Testing

In this study, eight hypotheses presented through structural equations using the maximum likelihood method are verified. First, the fit of the structural model is verified. Results of the verification are as follows: χ^2^ = 468.439, df = 160, *p* < 0.001, χ^2^/df = 2.928, RMSEA = 0.079, CFI = 0.956, and TLI = 0.948. Therefore, the fit of the structural model presented in this study is found to be at an appropriate level. Second, the influence of water conservation management and waste reduction measures on the social norms is reviewed to verify H1 and H2. Thus, water conservation management (β = 0.423, *p* < 0.01) is found to have a significant effect on the social norms, but waste reduction measures (β = 0.178, *p* > 0.05) have no significant effect on the social norms. To test H3 and H4, the impact of water conservation management and waste reduction measures on personal norms is investigated. As a result, both water conservation management (β = 0.202, *p* < 0.05) and waste reduction measures (β = 0.333, *p* < 0.01) are found to have a significant effect on personal norms. Next, to test H5–8, the impact of social norms and personal norms on willingness to pay and revisit intention is investigated. Consequently, the social and personal norms show a significant effect on the willingness to pay (β = 0.459, *p* < 0.01/β = 0.387, *p* < 0.01) and revisit intention (β = 0.433, *p* < 0.01/β = 0.307, *p* < 0.01). Therefore, of the eight hypotheses presented in this study, seven hypotheses except for H2 are accepted.

**Figure 1:**
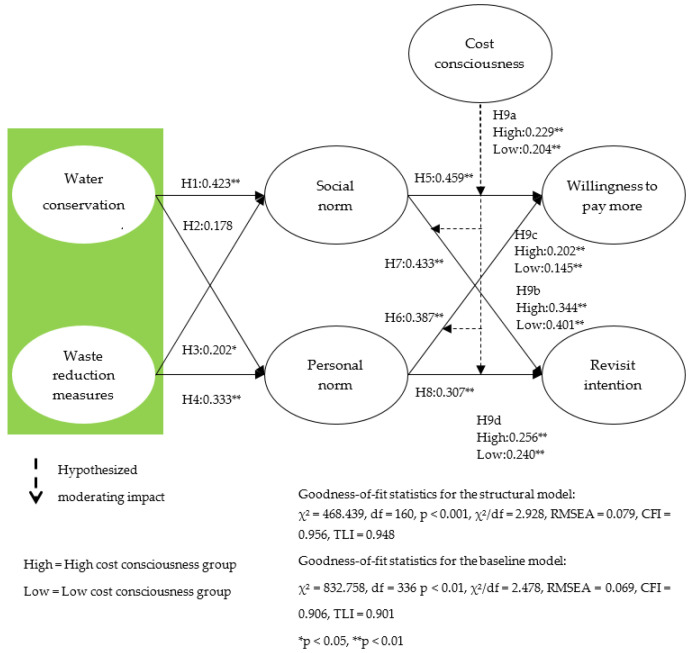
Structural model results and hypotheses testing.

In this study, a mediating framework is used to help understand the complex relationship of the proposed research model. That is, the indirect effect is verified using bootstrap. As a result of the analysis, water conservation management shows willingness to pay more (β water conservation management–social norm and personal norm–willingness to pay more = 0.273, *p* < 0.05) and revisit intention (β water conservation management–social norm and personal norm–revisit intention = 0.245, *p* < 0.05) shows a significant indirect effect. Likewise, waste reduction measures also have a set of willingness to pay more (β waste reduction measures–social norm and personal norm –willingness to pay more = 0.211, *p* < 0.05) and revisit intention (β waste reduction measures–social norm and personal norm–revisit intention = 0.180, *p* < 0.05) shows a significant indirect effect. Therefore, the mediating role of social norms and personal norms is proven within the theoretical framework presented in this study. Detailed results are shown in Table 2 and Figure 1.

### 4.3. Structural Invariance Model Results

An invariance test was conducted to verify the moderating role of cost consciousness in the relationship between social norms and personal norms on willingness to pay and revisit intention in the proposed research model. Thus, H9a–d are tested by dividing the cost consciousness into a high group (*n* = 68) and a low group (*n* = 243). The results of the analysis show that cost consciousness play a significant control role in the relationship between social norms’ willingness to pay (Δχ^2^(1) = 7.649, *p* < 0.01) and revisit intention (Δχ^2^(1) = 4.484, *p* < 0.05). However, in the relationship between personal norms and willingness to pay (Δχ^2^(1) = 1.852, *p* > 0.05) and revisit intention (Δχ^2^(1) = 0.525, *p* > 0.05), cost consciousness does not play a significant moderating role. Therefore, H9b and H9d are rejected and H9a and H9c are accepted. Detailed results are shown in Table 3 and Figure 1.

## 5. Discussion and Implication

This study aims to help us understand the effect of hotel eco-friendly activities on social and personal norms, willingness to pay more, and revisit intention. Specifically, the hotel’s eco-friendly activities were divided into water conservation and waste reduction measures, and the effects of these two variables on the social and personal norms were investigated. In addition, the effect of social and personal norms on willingness to pay more and revisit intention was investigated, and the moderating role of cost consciousness in the relationship between variables was also verified. To achieve the purpose of this study, an empirical analysis was conducted. It was found that all measurement items presented through the measurement model verification had an appropriate level of reliability and validity. The conceptual framework presented in this study through structural equation verification showed that hotels’ eco-friendly activities affect customer’s social and personal norms. The conceptual framework also positively forms the customer’s social and personal norms and the customer’s behavioral intention was well explained. In addition, a partial moderating role was confirmed in the relationship between social and personal norms, willingness to pay more, and revisit intention by examining the moderating role of cost awareness.

The results of the analysis of the eight hypotheses presented in this study are as follows. Water conservation management and waste reduction measures suggested as sub-factors of eco-friendly activities of hotels are found to have a positive effect on social and personal norms. These results affirm the importance of eco-friendly activities of hotels and further supported that they can serve as important clues to explain the process of enhancing hotel performance. Many studies also show that eco-friendly activities such as water conservation management and waste reduction measures form positive intentions of customers [5,15,16,18]. Therefore, it can be said that a hotel’s eco-friendly activities are important predictors that can form positive intentions and beliefs about the hotel. Next, both social and personal norms have a positive effect on willingness to pay more and intention to revisit. These results are consistent with the results of many previous studies that positive evaluations and perceptions on eco-friendly activities induce positive behaviors [19,20,21,26]. In other words, a hotel’s eco-friendly activities form the customer’s eco-friendly intention, which is a key factor that directly affects the hotel’s performance. Therefore, considering the results of the hypotheses tested in this study, it is inferred that hotels’ eco-friendly activities are very important and necessary strategies to improve the overall performance of the hotel.

As a result of examining the moderating role of cost awareness to test H9 of this study, it is found that there was a partial difference between the large group and the small group of cost awareness. Specifically, cost consciousness is confirmed to have a statistically significant moderating role in the relationship between social norms, willingness to pay more, and revisit intention, and a statistically significant moderating role is not found in the relationship between personal norms, willingness to pay more, and revisit intention. These results indicate that when the personal norms for eco-friendly activities are strong and have strong personal principles and beliefs, people will pay more for a hotel regardless of cost awareness and have a strong intention to revisit. As for the social norm, it is found that differences in customer behavior can appear according to differences in customer perception of cost. Such a result can be said to be theoretically very meaningful. Nevertheless, it is undoubtedly important to form both social and personal norms for eco-friendly activities in order to improve the performance of hotels.

This study extends existing studies by suggesting water conservation management and waste reduction measures as specific strategies for the hotel’s eco-friendly activities. In particular, it is a very meaningful discovery that personal norms can be strongly formed through the eco-friendly activities of hotels. Therefore, the results of this study successfully expanded the existing studies on the eco-friendly activities of hotels and successfully resolved the shortcomings. Based on the results of this study, hotel management can recognize the importance of the hotel’s eco-friendly activities and can establish various strategies to that end. More specifically, the management can introduce a state-of-the-art water quality control system for water conservation and waste reduction and provide eco-friendly products and services to customers. Thus, without using disposable products, eco-friendly posters and eco-friendly campaigns can be mobilized to induce eco-friendly activities of customers. It is necessary to clearly recognize that such an eco-friendly activity does not incur more costs. Instead, it is an investment that can ultimately maximize the hotel’s performance. 

## 6. Conclusions

Consumers’ interest in the environment continues to increase, and consumers have a favorable attitude toward a company’s eco-friendly activities. Therefore, the eco-friendly activities of hotels are classified into water conservation management and waste reduction measures, and its effect on social and personal norms and revisit intention is investigated. The empirical analysis conducted to achieve the purpose of this study shows that hotel eco-friendly activities had a positive effect on the social and personal norms, and that social and personal norms had a positive effect on willingness to pay more and revisit intention. Moreover, the moderating role of social and personal norms within the proposed theoretical framework are verified. It is found that cost consciousness plays a moderating role in the relationship between social norms, willingness to pay more, and revisit intention within the proposed theoretical framework. Thus, the purpose of this study is successfully achieved, and several meaningful implications are provided. 

This study has several limitations despite the various meaningful findings. First, as this study only mentions hotel-related activities, generalization to other industries is limited. Second, as the data used in the empirical analysis of this study were limited to Korean citizens, there is a limit to expanding it to other countries, ethnic groups, and continents. Third, as the respondents to the survey in this study were limited to hotel guests who have used hotels for the past year, the opinions of potential guests who have never used a hotel for eco-friendly activities were excluded. Lastly, it was not verified whether the respondents to the survey had ever stayed at an eco-friendly hotel, or whether they had any knowledge of the hotel’s eco-friendly activities. Therefore, future research could expand the research area to other industries, regions, and cultures, as well as studies targeting potential guests who have never used hotels.

## Figures and Tables

**Table 1:** Measurement model results and correlations.

	(1)	(2)	(3)	(4)	(5)	(6)	(7)
(1) WCM	1.000	–					
(2) WRM	0.611 ^a^ (0.373) ^b^	1.000					
(3) SN	0.552 (0.304)	0.510 (0.260)	1.000				
(4) PN	0.464 (0.215)	0.492 (0.242)	0.680 (0.462)	1.000			
(5) WP	0.534 (0.285)	0.493 (0.243)	0.574 (0.329)	0.583 (0.339)	1.000		
(6) RI	0.461 (0.212)	0.440 (0.193)	0.674 (0.454)	0.482 (0.222)	0.667 (0.444)	1.000	
(7) CC	0.518 (0.238)	0.537 (0.288)	0.605 (0.366)	0.610 (0.372)	0.658 (0.432)	0.623 (0.388)	1.000
Mean	5.614	5.569	5.586	5.618	5.890	6.131	5.979
SD	1.277	1.381	1.256	1.284	1.084	0.922	0.988
CR	0.937	0.896	0.838	0.891	0.889	0.920	0.878
AVE	0.788	0.684	0.636	0.732	0.727	0.793	0.706

Note. WCM: water conservation management, WRM: waste reduction measures, SN: social norm, PN: personal norm, WP: willingness to pay more, RI: revisit intention, CC: cost consciousness. Goodness-of-fit statistics for the measurement model: χ^2^ = 454.623, df = 209, *p* < 0.001, χ^2^/df = 2.175, RMSEA = 0.062, CFI = 0.970, TLI = 0.963. ^a^ Correlations, ^b^ Squared correlations.

**Table 2:** Structure model results.

Hypothesized Paths			Coefficients	*t*-Values
H1: WCM	→	SN	0.423	4.452 **
H2: WRM	→	SN	0.178	1.916
H3: WCM	→	PN	0.202	2.113 *
H4: WRM	→	PN	0.333	3.444 **
H5: SN	→	WP	0.459	4.448 **
H6: PN	→	WP	0.387	3.859 **
H7: SN	→	RI	0.433	3.671 **
H8: PN	→	RI	0.307	2.654 **
Indirect effect: β _WCM → SN & PN → WP_ = 0.273 * β _WCM → SN & PN → RI_ = 0.245 * β _WRM → SN & PN → WP_ = 0.211 * β _WRM → SN & PN → RI_ = 0.180 *			Explained variance: R^2^ (SN) = 0.261 R^2^ (PN) = 0.333 R^2^ (WP) = 0.675 R^2^ (RI) = 0.517	

* *p* < 0.05, ** *p* < 0.01. Note. WCM: water conservation management, WRM: waste reduction measures, SN: social norm, PN: personal norm, WP: willingness to pay more, RI: revisit intention, CC: cost consciousness. Goodness-of-fit statistics for the structural model: χ^2^ = 468.439, df = 160, *p* < 0.001, χ^2^/df = 2.928, RMSEA = 0.079, CFI = 0.956, TLI = 0.948.

**Table 3:** Results of invariance test for structural models.

Paths	High CC Group (*n* = 68)	Low CC Group (*n* = 243)	Baseline Model (Freely Estimated)	Nested Model (Constrained to Be Equal)
	β	β		
H9a: SN → WP	0.229 **	0.204 **	χ^2^ (336) = 832.758	χ^2^ (337) = 840.407 ^a^
H9b: PN → WP	0.344 **	0.401 **	χ^2^ (336) = 832.758	χ^2^ (337) = 834.610 ^b^
H9c: SN → RI	0.202 **	0.145 **	χ^2^ (336) = 832.758	χ^2^ (337) = 837.242 ^c^
H9c: PN → RI	0.256 **	0.240 **	χ^2^ (336) = 832.758	χ^2^ (337) = 833.283 ^d^
Chi-square test: ^a^ Δχ^2^ (1) = 7.649, *p* < 0.05 ^b^ Δχ^2^ (1) = 1.852, *p* > 0.05 ^c^ Δχ^2^ (1) = 4.484, *p* < 0.05 ^d^ Δχ^2^ (1) = 0.525, *p* > 0.05	Hypotheses testing: H9a: Supported H9b: Not supported H9c: Supported H9d: Not supported		Goodness-of-fit statistics for the baseline model: χ^2^ = 832.758, df = 336 *p* < 0.01, χ^2^/df = 2.478, RMSEA = 0.069, CFI = 0.906, TLI = 0.901 ** *p* < 0.01	

Note. WCM: water conservation management, WRM: waste reduction measures, SN: social norm, PN: personal norm, WP: willingness to pay more, RI: revisit intention, CC: cost consciousness.

## Data Availability

The dataset used in this research are available upon request from the corresponding author. The data are not publicly available due to restrictions i.e., privacy or ethical.
